# Aqua­{6,6′-dimeth­oxy-2,2′-[ethane-1,2-diylbis(nitrilo­methyl­idyne)]diphenolato-κ^4^ *O*,*N*,*N*′,*O*′}(formato-κ*O*)manganese(III) dihydrate

**DOI:** 10.1107/S1600536811017648

**Published:** 2011-05-14

**Authors:** See Mun Lee, Kong Mun Lo, Seik Weng Ng

**Affiliations:** a Department of Chemistry, University of Malaya, 50603 Kuala Lumpur, Malaysia

## Abstract

The Mn^III^ atom in the title complex, [Mn(C_18_H_18_N_2_O_4_)(CHO_2_)(H_2_O)]·2H_2_O, is *O*,*N*,*N*′,*O*′-chelated by the deproton­ated Schiff base; the four chelating atoms form an approximate square, with the O atoms of the water mol­ecule and the formate ion in axial positions above and below the square plane. Two metal-bearing mol­ecules are linked by an O—H_water_⋯O hydrogen bond about a center of inversion, generating a hydrogen-bonded dinuclear species; adjacent dinuclear units are linked through the lattice water mol­ecules, forming a three-dimensional network.

## Related literature

For related Mn^III^ compounds with the same Schiff base, see: Bermejo *et al.* (2007 ▶); Li *et al.* (2009 ▶); Zhang *et al.* (1999 ▶, 2000 ▶).
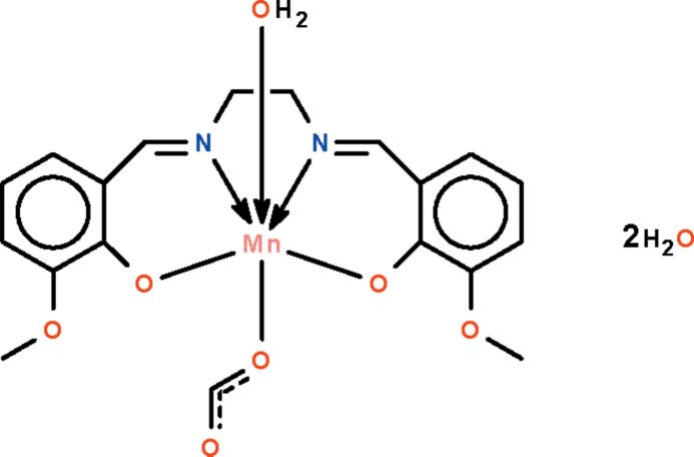


## Experimental

### 

#### Crystal data

[Mn(C_18_H_18_N_2_O_4_)(CHO_2_)(H_2_O)]·2H_2_O
*M* *_r_* = 480.35
Monoclinic, 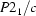

*a* = 11.5670 (2) Å
*b* = 19.9312 (3) Å
*c* = 8.7701 (1) Å
β = 96.859 (1)°
*V* = 2007.42 (5) Å^3^
*Z* = 4
Mo *K*α radiation
μ = 0.72 mm^−1^
*T* = 100 K
0.10 × 0.10 × 0.10 mm

#### Data collection

Bruker SMART APEX diffractometer
Absorption correction: multi-scan (*SADABS*; Sheldrick, 1996 ▶) *T*_min_ = 0.932, *T*_max_ = 0.932
18245 measured reflections
4600 independent reflections
3660 reflections with *I* > 2σ(*I*)
*R*_int_ = 0.054

#### Refinement

*R*[*F* ^2^ > 2σ(*F* ^2^)] = 0.067
*wR*(*F* ^2^) = 0.192
*S* = 1.12
4600 reflections
280 parameters
H-atom parameters constrained
Δρ_max_ = 1.67 e Å^−3^
Δρ_min_ = −0.96 e Å^−3^

### 

Data collection: *APEX2* (Bruker, 2009 ▶); cell refinement: *SAINT* (Bruker, 2009 ▶); data reduction: *SAINT*; program(s) used to solve structure: *SHELXS97* (Sheldrick, 2008 ▶); program(s) used to refine structure: *SHELXL97* (Sheldrick, 2008 ▶); molecular graphics: *X-SEED* (Barbour, 2001 ▶); software used to prepare material for publication: *publCIF* (Westrip, 2010 ▶).

## Supplementary Material

Crystal structure: contains datablocks global, I. DOI: 10.1107/S1600536811017648/bt5546sup1.cif

Structure factors: contains datablocks I. DOI: 10.1107/S1600536811017648/bt5546Isup2.hkl

Additional supplementary materials:  crystallographic information; 3D view; checkCIF report

## Figures and Tables

**Table 1:** Hydrogen-bond geometry (Å, °)

*D*—H⋯*A*	*D*—H	H⋯*A*	*D*⋯*A*	*D*—H⋯*A*
O1w—H11⋯O3^i^	0.84	2.01	2.730 (4)	143
O1w—H12⋯O2^i^	0.84	2.09	2.813 (4)	145
O2w—H21⋯O6	0.84	2.04	2.793 (5)	148
O2w—H22⋯O3w	0.84	1.96	2.762 (7)	161
O3w—H31⋯O2w^ii^	0.84	1.94	2.782 (7)	179

Symmetry codes: (i) 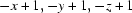; (ii) 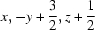.

**Fig. 1.**
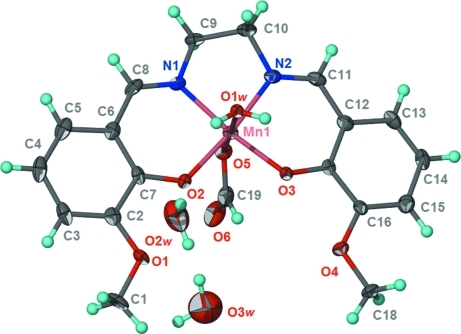
Anisotropic displacement ellipsoid plot (Barbour, 2001) of Mn(H2O)(CHO2)(C18H18N2O4).2H2O at the 70% probability level; hydrogen atoms are drawn as spheres of arbitrary radius.

## supplementary crystallographic information

### Comment

The 6,6'-dimethoxy-2,2'-[ethane-1,2-diylbis(nitrilomethylidyne)]diphenol Schiff base functions as a tetradentate dianionic ligand in a small number of manganese(III) derivatives (Bermejo *et al.*, 2007; Li *et al.* 2009; Zhang *et al.*, 1999, 2000). The counterion that balances the positive charge on the metal is involved in coordination. In the present study, the acetate portion of the manganese(III) acetate reactant undergoes carbon-carbon cleave to form a formate ion. The Mn^III^ atom in Mn(H_2_O)(CHO_2_)(C_18_H_18_N_2_O_4_)^.^2H_2_O (Scheme I) is *O*,*N*,*N*',*O*'-chelated by the deprotonated Schiff base; the four atoms involved in chelating form a square, above and below which are the O atoms of the water molecule and the formate ion (Fig. 1). Two molecules are linked by an O–H_water_···O hydrogen bond dinuclear species; adjacent dinuclear units are linked through the lattice water molecules, forming a three-dimensional network (Table 1).

### Experimental

*N,N*'-Ethylenebis(4-methoxysalicylindeneaminate) was prepared by reacting 0.2 ml (3.1 mmol) of ethylenediamine with 0.9 g (6 mmol) of 2-hydroxy-4-methoxybenzaldehyde in 50 ml of ethanol. An aqueous solution of 0.18 g (1 mmol) of manganese(II) nitrate was added to a hot methanol solution (100 ml) containing 0. 32 g (1 mmol) of the ligand and 0.28 g (2 mmol) of sodium acetate trihydrate. The immediate brown solution was refluxed for an hour, after which it was filtered. Slow evaporation of the filtrate gave brown crystals. Under the reaction conditions, the acetate ion used in the synthesis was converted to the formate ion in the product; the carbon-carbon bond cleavage was accompanied by oxidation of the Mn^II^ to Mn^III^.

### Refinement

Carbon- and oxygen-bound H-atoms were placed in calculated positions (C—H 0.95 to 0.98, O—H 0.84 Å) and were included in the refinement in the riding model approximation, with *U*(H) set to 1.2–1.5 times *U*_eq_(C). The water H atoms were placed on the basis of hydrogen bonding interactions. The O3w atom forms one only hydrogen bond; the O atom appears to be a little disordered but the disorder could not be modeled. The H31 atom is 1.99 Å from another H atom.

The final differerence Fourier map had a peak in the vicinity of Mn1.

### Crystal data

**Table:**

[Mn(C_18_H_18_N_2_O_4_)(CHO_2_)(H_2_O)]·2H_2_O	*F*(000) = 1000
*M**_r_* = 480.35	*D*_x_ = 1.589 Mg m^−^^3^
Monoclinic, *P*2_1_/*c*	Mo *K*α radiation, λ = 0.71073 Å
Hall symbol: -P 2ybc	Cell parameters from 5455 reflections
*a* = 11.5670 (2) Å	θ = 2.6–28.0°
*b* = 19.9312 (3) Å	µ = 0.72 mm^−^^1^
*c* = 8.7701 (1) Å	*T* = 100 K
β = 96.859 (1)°	Prism, brown
*V* = 2007.42 (5) Å^3^	0.10 × 0.10 × 0.10 mm
*Z* = 4	

### Data collection

**Table:**

Bruker SMART APEX diffractometer	4600 independent reflections
Radiation source: fine-focus sealed tube	3660 reflections with *I* > 2σ(*I*)
graphite	*R*_int_ = 0.054
ω scans	θ_max_ = 27.5°, θ_min_ = 2.1°
Absorption correction: multi-scan (*SADABS*; Sheldrick, 1996)	*h* = −15→15
*T*_min_ = 0.932, *T*_max_ = 0.932	*k* = −25→25
18245 measured reflections	*l* = −11→11

### Refinement

**Table:**

Refinement on *F*^2^	Primary atom site location: structure-invariant direct methods
Least-squares matrix: full	Secondary atom site location: difference Fourier map
*R*[*F*^2^ > 2σ(*F*^2^)] = 0.067	Hydrogen site location: inferred from neighbouring sites
*wR*(*F*^2^) = 0.192	H-atom parameters constrained
*S* = 1.12	*w* = 1/[σ^2^(*F*_o_^2^) + (0.0885*P*)^2^ + 6.8815*P*] where *P* = (*F*_o_^2^ + 2*F*_c_^2^)/3
4600 reflections	(Δ/σ)_max_ = 0.001
280 parameters	Δρ_max_ = 1.67 e Å^−^^3^
0 restraints	Δρ_min_ = −0.96 e Å^−^^3^

### Fractional atomic coordinates and isotropic or equivalent isotropic displacement parameters (Å^2^)

**Table:**

	*x*	*y*	*z*	*U*_iso_*/*U*_eq_	
Mn1	0.40763 (5)	0.60235 (3)	0.45697 (6)	0.01376 (19)	
O1	0.6169 (2)	0.53984 (14)	0.8928 (3)	0.0177 (6)	
O2	0.5069 (2)	0.58740 (13)	0.6460 (3)	0.0144 (5)	
O3	0.3207 (2)	0.52060 (13)	0.4678 (3)	0.0125 (5)	
O4	0.2108 (2)	0.42353 (13)	0.5778 (3)	0.0158 (6)	
O5	0.2946 (2)	0.65306 (13)	0.5659 (3)	0.0162 (6)	
O6	0.2078 (3)	0.65735 (18)	0.7803 (4)	0.0348 (8)	
O1W	0.5237 (2)	0.55239 (13)	0.3407 (3)	0.0126 (5)	
H11	0.5894	0.5451	0.3896	0.019*	
H12	0.4944	0.5155	0.3105	0.019*	
O2W	0.1084 (3)	0.7667 (2)	0.9154 (4)	0.0449 (10)	
H21	0.1510	0.7465	0.8592	0.067*	
H22	0.0809	0.7383	0.9721	0.067*	
O3W	−0.0064 (5)	0.6998 (3)	1.1285 (6)	0.0685 (14)	
H31	0.0274	0.7096	1.2158	0.103*	
H32	−0.0035	0.6581	1.1146	0.103*	
N1	0.4905 (3)	0.68849 (16)	0.4208 (4)	0.0136 (6)	
N2	0.3178 (3)	0.62522 (16)	0.2534 (4)	0.0143 (6)	
C1	0.6694 (4)	0.5152 (2)	1.0374 (5)	0.0246 (9)	
H1A	0.6264	0.4758	1.0668	0.037*	
H1B	0.6676	0.5502	1.1155	0.037*	
H1C	0.7504	0.5026	1.0294	0.037*	
C2	0.6672 (3)	0.5951 (2)	0.8350 (4)	0.0161 (8)	
C3	0.7689 (3)	0.6259 (2)	0.8983 (5)	0.0202 (8)	
H3	0.8092	0.6095	0.9916	0.024*	
C4	0.8127 (4)	0.6813 (2)	0.8249 (5)	0.0233 (9)	
H4	0.8836	0.7017	0.8672	0.028*	
C5	0.7536 (3)	0.7061 (2)	0.6929 (5)	0.0209 (9)	
H5	0.7838	0.7438	0.6445	0.025*	
C6	0.6482 (3)	0.67655 (19)	0.6269 (4)	0.0155 (7)	
C7	0.6036 (3)	0.61946 (19)	0.6976 (4)	0.0138 (7)	
C8	0.5872 (3)	0.70852 (19)	0.4923 (5)	0.0170 (8)	
H8	0.6216	0.7474	0.4542	0.020*	
C9	0.4287 (4)	0.72776 (19)	0.2943 (5)	0.0184 (8)	
H9A	0.4841	0.7575	0.2491	0.022*	
H9B	0.3683	0.7560	0.3333	0.022*	
C10	0.3726 (4)	0.6793 (2)	0.1728 (5)	0.0189 (8)	
H10A	0.3133	0.7029	0.1015	0.023*	
H10B	0.4322	0.6605	0.1127	0.023*	
C11	0.2202 (3)	0.59919 (19)	0.1976 (4)	0.0162 (7)	
H11A	0.1824	0.6176	0.1049	0.019*	
C12	0.1639 (3)	0.54401 (19)	0.2656 (4)	0.0143 (7)	
C13	0.0536 (3)	0.5237 (2)	0.1934 (5)	0.0186 (8)	
H13	0.0186	0.5475	0.1060	0.022*	
C14	−0.0036 (3)	0.4701 (2)	0.2477 (5)	0.0201 (8)	
H14	−0.0770	0.4566	0.1967	0.024*	
C15	0.0461 (3)	0.4352 (2)	0.3783 (5)	0.0186 (8)	
H15	0.0056	0.3986	0.4167	0.022*	
C16	0.1531 (3)	0.45387 (18)	0.4506 (4)	0.0132 (7)	
C17	0.2160 (3)	0.50826 (18)	0.3954 (4)	0.0116 (7)	
C18	0.1525 (4)	0.3688 (2)	0.6400 (5)	0.0195 (8)	
H18A	0.2013	0.3508	0.7297	0.029*	
H18B	0.1376	0.3335	0.5622	0.029*	
H18C	0.0783	0.3843	0.6709	0.029*	
C19	0.2604 (4)	0.6283 (2)	0.6891 (5)	0.0232 (9)	
H19	0.2783	0.5825	0.7102	0.028*	

### Atomic displacement parameters (Å^2^)

**Table:**

	*U*^11^	*U*^22^	*U*^33^	*U*^12^	*U*^13^	*U*^23^
Mn1	0.0155 (3)	0.0130 (3)	0.0126 (3)	−0.0006 (2)	0.0011 (2)	0.0015 (2)
O1	0.0182 (14)	0.0221 (14)	0.0117 (13)	−0.0003 (11)	−0.0031 (10)	0.0014 (11)
O2	0.0141 (13)	0.0138 (13)	0.0151 (13)	−0.0037 (10)	0.0006 (10)	0.0000 (10)
O3	0.0129 (12)	0.0119 (12)	0.0119 (12)	−0.0012 (10)	−0.0022 (10)	0.0012 (9)
O4	0.0173 (14)	0.0134 (13)	0.0163 (13)	−0.0013 (10)	0.0007 (10)	0.0039 (10)
O5	0.0158 (13)	0.0145 (13)	0.0187 (14)	0.0010 (10)	0.0040 (11)	−0.0008 (10)
O6	0.038 (2)	0.0346 (19)	0.0353 (19)	−0.0131 (15)	0.0206 (16)	−0.0151 (15)
O1W	0.0116 (12)	0.0134 (12)	0.0122 (12)	−0.0006 (9)	−0.0002 (10)	0.0005 (10)
O2W	0.042 (2)	0.054 (2)	0.039 (2)	0.0025 (18)	0.0034 (17)	−0.0162 (18)
O3W	0.069 (3)	0.066 (3)	0.069 (3)	−0.004 (3)	0.003 (3)	0.004 (3)
N1	0.0152 (15)	0.0119 (15)	0.0142 (15)	0.0004 (12)	0.0036 (12)	0.0025 (12)
N2	0.0170 (16)	0.0128 (15)	0.0134 (15)	0.0022 (12)	0.0023 (12)	0.0032 (12)
C1	0.027 (2)	0.036 (2)	0.0102 (18)	0.0038 (18)	−0.0022 (16)	0.0031 (16)
C2	0.0155 (18)	0.0182 (19)	0.0147 (18)	0.0015 (14)	0.0023 (14)	−0.0046 (14)
C3	0.0149 (19)	0.027 (2)	0.0182 (19)	0.0027 (16)	−0.0017 (15)	−0.0113 (16)
C4	0.0142 (19)	0.026 (2)	0.031 (2)	−0.0031 (16)	0.0043 (16)	−0.0138 (18)
C5	0.0153 (19)	0.0170 (19)	0.032 (2)	−0.0030 (15)	0.0089 (16)	−0.0082 (16)
C6	0.0132 (18)	0.0145 (18)	0.0191 (19)	0.0002 (14)	0.0033 (14)	−0.0051 (14)
C7	0.0120 (17)	0.0161 (18)	0.0136 (17)	0.0016 (13)	0.0026 (14)	−0.0066 (14)
C8	0.0199 (19)	0.0106 (17)	0.022 (2)	−0.0009 (14)	0.0104 (16)	−0.0005 (14)
C9	0.023 (2)	0.0146 (18)	0.0177 (19)	0.0004 (15)	0.0034 (16)	0.0062 (15)
C10	0.020 (2)	0.020 (2)	0.0164 (19)	−0.0007 (15)	0.0041 (15)	0.0060 (15)
C11	0.0169 (18)	0.0170 (18)	0.0143 (18)	0.0051 (14)	0.0003 (14)	0.0032 (14)
C12	0.0151 (18)	0.0158 (18)	0.0119 (17)	0.0023 (14)	0.0008 (14)	−0.0010 (14)
C13	0.0157 (19)	0.023 (2)	0.0164 (18)	0.0043 (15)	−0.0025 (15)	−0.0015 (15)
C14	0.0141 (18)	0.026 (2)	0.020 (2)	−0.0025 (16)	−0.0020 (15)	−0.0052 (16)
C15	0.0154 (19)	0.021 (2)	0.0200 (19)	−0.0018 (15)	0.0029 (15)	−0.0021 (15)
C16	0.0151 (18)	0.0140 (17)	0.0103 (16)	−0.0005 (14)	0.0010 (13)	−0.0016 (13)
C17	0.0128 (17)	0.0111 (16)	0.0113 (16)	0.0013 (13)	0.0027 (13)	−0.0026 (13)
C18	0.025 (2)	0.0148 (18)	0.0192 (19)	−0.0049 (15)	0.0041 (16)	0.0028 (15)
C19	0.021 (2)	0.027 (2)	0.022 (2)	−0.0087 (17)	0.0062 (17)	−0.0055 (17)

### Geometric parameters (Å, °)

**Table:**

Mn1—O3	1.923 (3)	C3—C4	1.403 (6)
Mn1—O2	1.924 (3)	C3—H3	0.9500
Mn1—O5	1.985 (3)	C4—C5	1.364 (6)
Mn1—N2	2.008 (3)	C4—H4	0.9500
Mn1—N1	2.010 (3)	C5—C6	1.414 (5)
Mn1—O1W	2.040 (3)	C5—H5	0.9500
O1—C2	1.371 (5)	C6—C7	1.422 (5)
O1—C1	1.427 (5)	C6—C8	1.449 (6)
O2—C7	1.321 (4)	C8—H8	0.9500
O3—C17	1.322 (4)	C9—C10	1.525 (6)
O4—C16	1.370 (4)	C9—H9A	0.9900
O4—C18	1.426 (5)	C9—H9B	0.9900
O5—C19	1.293 (5)	C10—H10A	0.9900
O6—C19	1.208 (5)	C10—H10B	0.9900
O1W—H11	0.8400	C11—C12	1.443 (5)
O1W—H12	0.8400	C11—H11A	0.9500
O2W—H21	0.8400	C12—C13	1.414 (5)
O2W—H22	0.8399	C12—C17	1.415 (5)
O3W—H31	0.8401	C13—C14	1.371 (6)
O3W—H32	0.8399	C13—H13	0.9500
N1—C8	1.279 (5)	C14—C15	1.403 (6)
N1—C9	1.471 (5)	C14—H14	0.9500
N2—C11	1.285 (5)	C15—C16	1.373 (5)
N2—C10	1.473 (5)	C15—H15	0.9500
C1—H1A	0.9800	C16—C17	1.423 (5)
C1—H1B	0.9800	C18—H18A	0.9800
C1—H1C	0.9800	C18—H18B	0.9800
C2—C3	1.383 (5)	C18—H18C	0.9800
C2—C7	1.421 (5)	C19—H19	0.9500
			
O3—Mn1—O2	95.04 (11)	C5—C6—C8	117.6 (4)
O3—Mn1—O5	91.70 (11)	C7—C6—C8	122.7 (3)
O2—Mn1—O5	91.35 (11)	O2—C7—C2	117.2 (3)
O3—Mn1—N2	91.04 (12)	O2—C7—C6	125.1 (3)
O2—Mn1—N2	173.53 (12)	C2—C7—C6	117.7 (3)
O5—Mn1—N2	90.68 (12)	N1—C8—C6	125.1 (3)
O3—Mn1—N1	173.52 (12)	N1—C8—H8	117.5
O2—Mn1—N1	91.25 (12)	C6—C8—H8	117.5
O5—Mn1—N1	89.71 (12)	N1—C9—C10	108.5 (3)
N2—Mn1—N1	82.62 (13)	N1—C9—H9A	110.0
O3—Mn1—O1W	89.43 (11)	C10—C9—H9A	110.0
O2—Mn1—O1W	89.70 (11)	N1—C9—H9B	110.0
O5—Mn1—O1W	178.39 (11)	C10—C9—H9B	110.0
N2—Mn1—O1W	88.15 (12)	H9A—C9—H9B	108.4
N1—Mn1—O1W	89.05 (12)	N2—C10—C9	107.4 (3)
C2—O1—C1	116.8 (3)	N2—C10—H10A	110.2
C7—O2—Mn1	127.9 (2)	C9—C10—H10A	110.2
C17—O3—Mn1	126.0 (2)	N2—C10—H10B	110.2
C16—O4—C18	116.2 (3)	C9—C10—H10B	110.2
C19—O5—Mn1	119.6 (3)	H10A—C10—H10B	108.5
Mn1—O1W—H11	116.0	N2—C11—C12	124.8 (3)
Mn1—O1W—H12	108.7	N2—C11—H11A	117.6
H11—O1W—H12	108.4	C12—C11—H11A	117.6
H21—O2W—H22	108.4	C13—C12—C17	119.4 (3)
H31—O3W—H32	110.0	C13—C12—C11	117.5 (3)
C8—N1—C9	121.4 (3)	C17—C12—C11	123.1 (3)
C8—N1—Mn1	126.5 (3)	C14—C13—C12	121.0 (4)
C9—N1—Mn1	112.1 (2)	C14—C13—H13	119.5
C11—N2—C10	121.4 (3)	C12—C13—H13	119.5
C11—N2—Mn1	125.8 (3)	C13—C14—C15	120.1 (4)
C10—N2—Mn1	112.7 (2)	C13—C14—H14	119.9
O1—C1—H1A	109.5	C15—C14—H14	119.9
O1—C1—H1B	109.5	C16—C15—C14	120.1 (4)
H1A—C1—H1B	109.5	C16—C15—H15	119.9
O1—C1—H1C	109.5	C14—C15—H15	119.9
H1A—C1—H1C	109.5	O4—C16—C15	125.6 (3)
H1B—C1—H1C	109.5	O4—C16—C17	113.2 (3)
O1—C2—C3	125.9 (4)	C15—C16—C17	121.2 (4)
O1—C2—C7	112.8 (3)	O3—C17—C12	124.8 (3)
C3—C2—C7	121.2 (4)	O3—C17—C16	117.1 (3)
C2—C3—C4	120.1 (4)	C12—C17—C16	118.1 (3)
C2—C3—H3	119.9	O4—C18—H18A	109.5
C4—C3—H3	119.9	O4—C18—H18B	109.5
C5—C4—C3	120.2 (4)	H18A—C18—H18B	109.5
C5—C4—H4	119.9	O4—C18—H18C	109.5
C3—C4—H4	119.9	H18A—C18—H18C	109.5
C4—C5—C6	121.1 (4)	H18B—C18—H18C	109.5
C4—C5—H5	119.5	O6—C19—O5	126.8 (5)
C6—C5—H5	119.5	O6—C19—H19	116.6
C5—C6—C7	119.6 (4)	O5—C19—H19	116.6
			
O3—Mn1—O2—C7	166.9 (3)	C3—C2—C7—O2	−179.4 (3)
O5—Mn1—O2—C7	−101.3 (3)	O1—C2—C7—C6	−179.7 (3)
N1—Mn1—O2—C7	−11.5 (3)	C3—C2—C7—C6	0.1 (5)
O1W—Mn1—O2—C7	77.5 (3)	C5—C6—C7—O2	−179.5 (3)
O2—Mn1—O3—C17	159.0 (3)	C8—C6—C7—O2	3.6 (6)
O5—Mn1—O3—C17	67.5 (3)	C5—C6—C7—C2	1.1 (5)
N2—Mn1—O3—C17	−23.2 (3)	C8—C6—C7—C2	−175.8 (3)
O1W—Mn1—O3—C17	−111.4 (3)	C9—N1—C8—C6	173.9 (3)
O3—Mn1—O5—C19	46.9 (3)	Mn1—N1—C8—C6	−8.3 (6)
O2—Mn1—O5—C19	−48.1 (3)	C5—C6—C8—N1	−179.1 (4)
N2—Mn1—O5—C19	138.0 (3)	C7—C6—C8—N1	−2.1 (6)
N1—Mn1—O5—C19	−139.4 (3)	C8—N1—C9—C10	143.4 (4)
O2—Mn1—N1—C8	12.7 (3)	Mn1—N1—C9—C10	−34.8 (4)
O5—Mn1—N1—C8	104.1 (3)	C11—N2—C10—C9	142.6 (4)
N2—Mn1—N1—C8	−165.2 (3)	Mn1—N2—C10—C9	−34.4 (4)
O1W—Mn1—N1—C8	−76.9 (3)	N1—C9—C10—N2	44.2 (4)
O2—Mn1—N1—C9	−169.2 (3)	C10—N2—C11—C12	176.8 (3)
O5—Mn1—N1—C9	−77.9 (3)	Mn1—N2—C11—C12	−6.5 (6)
N2—Mn1—N1—C9	12.8 (2)	N2—C11—C12—C13	176.8 (4)
O1W—Mn1—N1—C9	101.1 (2)	N2—C11—C12—C17	−6.0 (6)
O3—Mn1—N2—C11	17.3 (3)	C17—C12—C13—C14	0.3 (6)
O5—Mn1—N2—C11	−74.5 (3)	C11—C12—C13—C14	177.6 (4)
N1—Mn1—N2—C11	−164.1 (3)	C12—C13—C14—C15	1.1 (6)
O1W—Mn1—N2—C11	106.6 (3)	C13—C14—C15—C16	−1.1 (6)
O3—Mn1—N2—C10	−165.8 (3)	C18—O4—C16—C15	−1.1 (5)
O5—Mn1—N2—C10	102.5 (3)	C18—O4—C16—C17	179.6 (3)
N1—Mn1—N2—C10	12.9 (3)	C14—C15—C16—O4	−179.4 (4)
O1W—Mn1—N2—C10	−76.4 (3)	C14—C15—C16—C17	−0.3 (6)
C1—O1—C2—C3	5.3 (5)	Mn1—O3—C17—C12	19.2 (5)
C1—O1—C2—C7	−175.0 (3)	Mn1—O3—C17—C16	−162.5 (2)
O1—C2—C3—C4	178.3 (4)	C13—C12—C17—O3	176.7 (3)
C7—C2—C3—C4	−1.5 (6)	C11—C12—C17—O3	−0.4 (6)
C2—C3—C4—C5	1.6 (6)	C13—C12—C17—C16	−1.6 (5)
C3—C4—C5—C6	−0.4 (6)	C11—C12—C17—C16	−178.8 (3)
C4—C5—C6—C7	−1.0 (6)	O4—C16—C17—O3	2.4 (5)
C4—C5—C6—C8	176.1 (4)	C15—C16—C17—O3	−176.8 (3)
Mn1—O2—C7—C2	−174.6 (2)	O4—C16—C17—C12	−179.1 (3)
Mn1—O2—C7—C6	6.0 (5)	C15—C16—C17—C12	1.7 (5)
O1—C2—C7—O2	0.9 (5)	Mn1—O5—C19—O6	167.1 (4)

### Hydrogen-bond geometry (Å, °)

**Table:**

*D*—H···*A*	*D*—H	H···*A*	*D*···*A*	*D*—H···*A*
O1w—H11···O3^i^	0.84	2.01	2.730 (4)	143
O1w—H12···O2^i^	0.84	2.09	2.813 (4)	145
O2w—H21···O6	0.84	2.04	2.793 (5)	148
O2w—H22···O3w	0.84	1.96	2.762 (7)	161
O3w—H31···O2w^ii^	0.84	1.94	2.782 (7)	179

Symmetry codes: (i) −*x*+1, −*y*+1, −*z*+1; (ii) *x*, −*y*+3/2, *z*+1/2.
